# Affections Determined by the Wisdom Teeth

**Published:** 1857-07

**Authors:** Henry Van Holsbick

**Affiliations:** late resident of the Hospital.


### ARTICLE V.

*Affections Determined by the Wisdom Teeth.* By Dr. Henry Van Holsbick, late resident of the Hospital. Translated from the French, from L'Art Dentaire, for the Am. Jour. of Dental Science.

The teeth at this period of their eruption or later, when they deviate from their normal position, frequently engender very serious affections, for the removal of which the most energetic and best directed means often prove unavailing.

That such should be the case is not astonishing, for it must be confessed that the pathology of the teeth is very little known, and it is thought to be of so little importance to examine these organs that when it becomes necessary to establish a diagnosis and institute treatment for diseases to which they are daily subject, we are at a loss to know what course to pursue.*

*That such is the case on the continent of Europe may be true, but the knowledge of the teeth is greater and their diseases are certainly much better understood in the United States and England.—*Translator.*

In the mean time we have a rule in medicine which we too often unfortunately neglect to apply, which teaches us that in order to employ a remedy successfully and obtain a complete and definite cure, it is first necessary to ascertain the cause, to make a first application of it, and do what is to be done, in the speediest and most effective manner.

These few preliminaries being established, we do not propose here to give a complete table of all the diseases to which the teeth are liable, but shall at once proceed to notice, and in a very brief manner, the morbid effects which máy be caused by the wisdom teeth, especially those of the lower jaw.

These effects result from the evolution of these teeth or are developed later as a consequence of disease in them, or from a normal position.

The wisdom teeth in their passage often encounter great resistance from the gum. The extent of these effects are determined by the age of the individual at the time of their evolution, being more strongly marked as he is advanced. It is owing to the resistance affected by the gums that those frightful convulsions so frequently seen among infants is attributable; it is still more to this that those nervous affections, sometimes so serious, met with in adults, are to be ascribed.

When the gum resists the passage of the tooth, the portion which covers it become more or less thickened.

It is then followed by tumefaction of the face, which occurs an indefinite number of times, and is always attended with the most intolerable pain. But the effects thus engendered do not end here; for the thickened gum over the tooth being kept in a state of permanent irritation by the movement of the jaws, extends to the tongue, that becomes the seat of more or less acute inflammation, which is propagated to the veil of the palate and often gives rise to enlargement of the tonsils. In this case the gum is red and swollen. It is at the same time hot. The action of mastication is difficult and painful.

What is necessary to be done under these circumstances? The gums should at once be incised. The incision should be long and deep, and a dossil of lint inserted in the wound over the tooth to separate the lips of it from each other. A simple incision does not always relieve the difficulty; sometimes it is necessary to extract the tooth, if the operation be practicable, or the corresponding upper tooth, or if more advantageous the adjoining one.

However, it is better to confine ourselves to a single incision of the gum.

The wisdom tooth is sometimes arrested in its progress by the narrowness of the space between the second molar aad the base of the coronoid process. In this case the patient is constantly exposed to swelling of the gum, so that it is often difficult for him to open his mouth. If this difficulty is not discovered in time and promptly prevented, by the removal of the second molar which opposes the passage of the tooth, the contiguous parts become the seat of active inflammation. The gum in this case soon suppurates, the socket and base of the coronoid process becomes implicated and carious, fistulas are established and sequestra, in greater or less number, escape from them. In a word, these are the effects which result when the case is left to itself. The cure is often protracted for a long time.

When relief is sought before the jaw has become too firmly closed, the wisdom tooth, if possible, should be immediately extracted or the second molar removed. This done, the morbid phenomena which have been developed will cease to exist.

But as a general thing, surgical aid is not sought until a period when an examination of the mouth is scarcely possible; under these circumstances appropriate mechanical means should be employed for gradually overcoming the existence of the masticatory muscles.

As soon as the jaws are sufficiently separated we should proceed in the manner as already indicated.

The wisdom teeth sometimes deviate in their growth from their normal direction; they may, in this case, become a source of serious trouble.

They sometimes assume an oblique direction from behind forward, pressing against the second molars which prevents them from coming through the gums. This causes acute pain in all the teeth of the affected side, but which, nevertheless, is not equal in intensity to ordinary tooth-ache. If the cause of this suffering is not detected, as frequently happens, and promptly treated, the severity of it increases, and the health of the patient is sometimes seriously affected by it.

When this happens the second molar should be extracted without delay.

If the wisdom tooth inclines inwards, facing the tongue, as is often the case, ulcers will be produced on this organ, which, being similar to those that occur in syphilis, are often attributed to it. These ulcers render the movements of the tongue more or less painful. Mastication is also so painful that the patient frequently prefers to fast than to endure it. To obtain a cure, it is necessary in the first place to extract the tooth which has given rise to them, and then to treat them as we would simple ulcers in other parts of the body.

The tooth is sometimes broken off in the operation, but the portion that remains is not in a condition to irritate the tongue.

The wisdom tooth sometimes comes out outwardly and imbeds itself in the substance of the cheek where it makes an ulcerous excavation. The patient in this case is subject to continual swelling of the face. A hard and painful projection is seen upon the cheek immediately over the part affected on the inside, painful to pressure. The remedial indication consists in the immediate removal of the tooth, but the extraction of this tooth is not always easy, for often the swelling of the gum and internal part of the cheek oppose an insurmountable obstacle. In this case, the first of all, it is necessary to dissipate the inflammation and isolate the tooth from the cheek. When the inflammation is overcome and the patient can open his mouth sufficiently, the tooth should be removed with such instrument as can be most easily applied to it.

These outward deviations of the wisdom teeth are of frequent occurrence, but fortunately, in very many cases, they are confined to pinching certain portions of the cheek during mastication, and do not really incommode the patient except when the tooth is decayed and presents asperities which excoriate the neighboring soft parts.

In such cases it is necessary to extract the tooth.

We add, in conclusion, two very important cases which appears to us to be worthy of the notice of the practitioner.

*Case* 1. In the month of July, 1856, M. N., a custom house officer, consulted me for an ulcer of the tongue, from which he had suffered much. Mastication was difficult and the movements of the tongue were extremely painful. He had had a chancre in the mean time and believed the ulcer had been brought on by syphilis.

An attentive exploration of the mouth at once convinced me that the ulcer for which he had sought my skill, was produced by no other cause than an inward deviation of the wisdom tooth, the decayed crown of which presented numerous asperities. I immediately proposed the extraction of the tooth and cauterized the ulcer with a pencil of nitrate of silver, in order to reduce it to the state of a simple wound. Six days later, M. N., was relieved from the cruel affection which had tormented him for so many months.

*Case* 2. In the month of December, 1856, my services were called into requisition for a woman, named Eliza G., aged twenty-six years.

For two or three months this person was affected with a large swelling of the left cheek. The wisdom tooth had deviated outwardly and the cheek presented in the corresponding part an ulcerous excavation of the size of the fiftieth part of a centime. The bottom had a grayish appearance, the sides were hard and uneven. I confined myself for the first few days to the reduction of the inflammation which existed and contented myself by separating the tooth from the cheek by means of a linen cushion sufficiently thick to receive the crown. At the end of the third day the inflammation was dissipated and she was enabled to open her mouth sufficiently to permit the extraction of the tooth; then I cauterized the ulcer with nitrate of silver. At the expiration of five days a simple wound only existed, which, in a short time completely cicatrized, and the projection on the cheek disappeared. A complete cure was effected.

